# DNA Homologous Recombination Factor SFR1 Physically and Functionally Interacts with Estrogen Receptor Alpha

**DOI:** 10.1371/journal.pone.0068075

**Published:** 2013-07-09

**Authors:** Yuxin Feng, David Singleton, Chun Guo, Amanda Gardner, Suresh Pakala, Rakesh Kumar, Elwood Jensen, Jinsong Zhang, Sohaib Khan

**Affiliations:** 1 Department of Cancer Biology, College of Medicine, University of Cincinnati, Cincinnati, Ohio, United States of America; 2 Department of Biochemistry and Molecular Biology, George Washington University School of Medicine, Washington, DC, United States of America; 3 Division of Experimental Hematology and Cancer Biology, Cincinnati Children’s Hospital Medical Center, Cincinnati, Ohio, United States of America; Karolinska Institutet, Sweden

## Abstract

Estrogen receptor alpha (ERα), a ligand-dependent transcription factor, mediates the expression of its target genes by interacting with corepressors and coactivators. Since the first cloning of SRC1, more than 280 nuclear receptor cofactors have been identified, which orchestrate target gene transcription. Aberrant activity of ER or its accessory proteins results in a number of diseases including breast cancer. Here we identified SFR1, a protein involved in DNA homologous recombination, as a novel binding partner of ERα. Initially isolated in a yeast two-hybrid screen, the interaction of SFR1 and ERα was confirmed in vivo by immunoprecipitation and mammalian one-hybrid assays. SFR1 co-localized with ERα in the nucleus, potentiated ER’s ligand-dependent and ligand-independent transcriptional activity, and occupied the ER binding sites of its target gene promoters. Knockdown of SFR1 diminished ER’s transcriptional activity. Manipulating SFR1 expression by knockdown and overexpression revealed a role for SFR1 in ER-dependent and -independent cancer cell proliferation. SFR1 differs from SRC1 by the lack of an intrinsic activation function. Taken together, we propose that SFR1 is a novel transcriptional modulator for ERα and a potential target in breast cancer therapy.

## Introduction

Estrogen signaling is mediated by two nuclear receptors, ERα and ERβ, which regulate a broad range of biological processes (Reviewed in [1], [2], [3]). Like other nuclear hormone receptors, ERα contains an N-terminal transactivation domain (AF1), a DNA binding domain, a hinge region and a ligand binding domain overlapping with the second transactivation domain (AF2). In the absence of estradiol, ERα is normally present in the nucleus as complex with heat shock proteins. Upon binding to estradiol, heat shock proteins are released, and ERα undergoes conformational changes, dimerizes and binds to the estrogen receptor responsive elements (EREs) of the target genes. This initiates recruitment of a number of transcriptional accessory proteins known as coactivators. The complex formation on the ERE facilitates the initiation of target gene transcription in responsive tissues to promote growth and proliferation [4].

In the absence of ligands, ERα (henceforth referred to as ER or ERα) is bound by corepressors including N-CoR and SMRT [5], through the corepressor’s CoRNR box motifs (LXXIXXXL) [6]. Ligand binding promotes a conformational change in ER most notably in the helix 12 region. Subsequently, a binding pocket, composed of helices 3, 4, 5 and the re-folded helix 12, is generated to attract the LXXLL motifs (NR-boxes) of coactivators [7], [8].

Nuclear receptor coactivators contribute to ERα-dependent gene transcription in different fashions, including acetylating and methylating histone tails, remodeling chromatin structure, recruiting and modifying other coactivators, and bridging the general transcription machinery with the nuclear receptors (reviewed in [4], [9]). Thus far, six groups of nuclear receptor coactivators have been characterized, including: i) p160 family coactivators (SRC1, SRC2/TIF2 and SRC3/AIB1), which directly bind to liganded ER and recruit additional coactivators such as CBP/p300 and CARM1 [10]; ii) CBP/p300 family members, acetylating p160 coactivators and histones, are responsible for the quick dispersal of the coactivator complex [11], [12]; iii) DRIP205/TRAP220/MED1, which resides in the human mediator complex, thus anchoring the general transcription machinery on the liganded nuclear receptors [13]; iv) subunits of the SWI/SNF chromatin remodeling complexes, including BAF57, Brm and Brg-1 [14], which help to remodel the chromatin to increase the accessibility for transcriptional factors/cofactors; v) non-p160 family proteins, including some co-factors, such as RIP140, TIF1 and ARA70, which can function as coactivators or corepressors depending on the promoter and receptor contexts [4]; and vi) the so-called secondary coactivators, including CoCoA and GAC63, which bind to p160 family members but do not directly interact with NRs [15], [16].

The classical corepressors and coactivators bind to ER in the strict ligand-independent and ligand-dependent manners. In this work, we considered the possibility of the existence of novel ER-interacting proteins that may bind to ER in manners different from that of classic corepressors and coactivators. To this end, an unbiased yeast two-hybrid screen was conducted to identify novel ER-interacting proteins, utilizing the CDEF domains of human ERα as bait, both in the absence and presence of ER ligands. We identified SFR1, a subunit of the Swi5-SFR1 (Mei5) complex involved in DNA recombination and repair [17], [18], [19], as a novel binding partner of ERα. SFR1 enhances ER’s ligand-independent and ligand-dependent transcriptional activity and also promotes breast cancer cell proliferation. SFR1, however, does not harbor an intrinsic activation function and shares no homology with known coactivators. We propose that SFR1 is a unique transcriptional modulator of ER that can facilitate both ligand-independent and ligand-dependent transcription. Importantly, our results also categorize SFR1 into the group of proteins that regulate both transcription and DNA repair.

## Results

### Yeast Two-hybrid Screen Identifies SFR1 Protein as an ERα-interacting Partner

**Figure 1:**
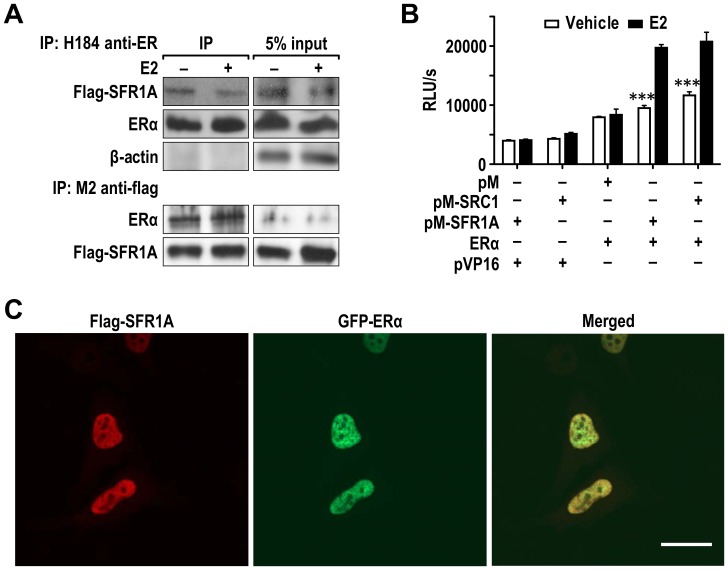
SFR1-A interacts with ERα in mammalian cells and colocalized with ERα in cell nucleus. (A) FLAG-SFR1-A and ER were co-transfected into Ishikawa cells. Co-IP experiments were performed with anti-ERα and anti-FLAG antibodies in Ishikawa cells in the presence and absence of E2. (B) The interaction of ERα and SFR1-A was further analyzed with mammalian one-hybrid experiments in Ishikawa cells. pM vector, pM-SFR1-A (SFR1-A) and pM-SRC1 were used as baits. Cells co-transfected with pM-SRC1 NR-box (SRC1) and pCMV5-ERα were used as positive control. (n = 4, p<0.005) (C) The localization of SFR1A and ERα was evaluated with immunefluorescent staining. HeLa cells were co-transfected with FLAG-SFR1-A and GFP-ERα. 24 hours after the transfection, cells were treated with E2 or vehicle overnight. The cells were fixed and stained with M2 anti-FLAG antibody and Alexa Fluor 568 anti-mouse secondary antibody. Immunostained cells were photographed using a Zeiss LSM510 confocal microscope with a 63X Zeiss objective. Scale bar = 20 µm. The error bars indicate standard deviations. * = P<0.05; ** = P<0.01; *** = P<0.005.

To identify novel co-factors of ERα, a yeast two-hybrid screen was performed using a human mammary gland cDNA library with ERα CDEF domain as the bait in the presence and absence of estrogen. A number of known and novel proteins were identified, including the p160 family member SRC1 [10] and the transcription factor Stat3 [20]. We focused on a novel protein coded by a gene on chromosome 10q25.1 (designated C10ORF78, accession no. NM_001002759). Coincidently, another group recently identified C10ORF78 as the human counterpart of yeast SFR1 (Mei1), a protein in the DNA homologous recombination pathway [17]. Although there are three putative isoforms named SFR1-A, -B, and -C based on the length of the predicted proteins, the longest isoform, which codes 307aa, should represent the true transcript in the cells (Figure S1A). Thus, western blot analysis showed a consistent 37 kDa band of FLAG-SFR1 protein across multiple cell types (Figure 1A). In addition, an independent study showed that mouse SFR1 is a 303 amino acid protein [18]. The deduced SFR1 protein is conserved in vertebrates, including mammals, chicken, zebra fish and Xenopus (Figure S1). From amino acid 67–307, about 74% of amino acids are identical among human, chimpanzee, monkey and dog. About 76% of amino acids are conserved among all 5 mammalian species (Figure S1B).

The sequence of SFR1 reflects its unique role as a nuclear receptor coregulator. At the conserved C-terminal region, SFR1 contains sequences (ΦXXΦXXXΦ, Φ; hydrophobic amino acids) similar to the CoRNR-box of corepressors and another sequence similar to the NR-box (ΦXXΦΦ, Φ; hydrophobic amino acids) of coactivators (Figure S1A-B). Supporting the notion that these motifs might mediate the interaction of SFR1 with nuclear receptors and, hence, carry functional importance [8], they are completely conserved among the different mammal species of SFR1 proteins. SFR1 also contains a putative coiled-coil region. Coiled-coil domains mediating protein-protein and/or protein-DNA interactions have been found in nuclear receptor coactivators [21], [22]. For example, CoCo-A, a secondary coactivator containing a coiled-coil domain, was found to enhance ER and AHR transcriptional regulatory activity [16]. Finally, although a nuclear localization signal was not identified, several sub-cellular localization prediction programs (WoLFPSORT, LOCtree and SignalP3.0) predicted SFR1 as a non-secretory protein localized in the cell nucleus, containing no DNA binding domain (data not shown). Taken together, the domains within SFR1 are indicative of hormone-receptor binding with nuclear function, consistent with its nuclear receptor coregulator activity.

### SFR1 mRNA is Expressed in Human Breast Cancer Cells

The information from GenBank indicated that human SFR1 mRNA was detected in multiple sex hormone-responsive tissues including mammary gland, male and female reproductive systems and prostate. Mouse SFR1 mRNA was detected in the mammary tumor of MMTV-int1 transgenic mice, suggesting possible involvement of SFR1 in mammary tumorigenesis. To address the role of SFR1 in ER-positive breast cancer cells, we examined the expression of SFR1 in several human cancer cell lines. SFR1 mRNA was detected in ERα-positive MCF7 breast cancer cells as well as Ishikawa endometrial adenocarcinoma cells (Figure S1C). The SFR1 transcripts were also detected in human skeletal muscle and fetal brain. The overlapping expression of SFR1 with ERα is consistent with their functional interaction in breast cancer cells.

### SFR1 Interacts with ERα in Estrogen-sensitive Ishikawa Cell Line Derived from Human Endometrial Cancer Cells

To confirm the interaction of SFR1 with ERα in mammalian cells, the cDNA of SFR1 was cloned into p3XFLAG-CMV-7.1 mammalian expression vector and transfected into Ishikawa cells (a cell line derived from human endometrial cancer). As predicted, a 37 kDa band was detected with anti-FLAG M2 antibody (Sigma), indicating that the fusion protein was expressed in mammalian cells (see below Figure 1A). We then examined the interaction between ERα and SFR1 proteins by reciprocal co-immunoprecipitation (Co-IP) assays in Ishikawa cells. Consistent with the yeast two-hybrid data, immunoprecipitation with both anti-FLAG and anti-ERα antibody showed that ERα and SFR1 can pull each other down (Figure 1A), supporting the notion that ER and SFR1 form a physical complex *in vivo*. To determine whether SFR1 can bind to other nuclear receptors, Co-IP experiments were performed with protein extracts from Ishikawa cells co-transfected with androgen receptor (AR) and SFR1. Similar to ER and SFR1 interaction, AR associated with SFR1 both in the absence and presence of dihydroxy-testosterone (DHT) (data not shown).

Next, we tested whether the biochemical interaction between ER and SFR1 can be recapitulated by the functional mammalian one-hybrid assay. The SFR1 cDNA was subcloned into the pM vector as bait. This fuses SFR1 with the first 147 amino acids of the Gal4 DNA-binding domain. The SRC1 cDNA fragment containing NR-boxes (SRC1-NR) known to interact with ERα was similarly fused to Gal4-DBD and used as a positive control. In the absence of ERα, neither SFR1 nor SRC1-NR, as a Gal4-fusion protein, was capable of activating luciferase reporter expression beyond the basal level. Co-expression of ER allowed both Gal4-SFR1 and Gal4-SRC1-NR to respond to estradiol (E2) stimulation (Figure 1B) by a similar magnitude. These results are consistent with a functional, intracellular binding between ER and SFR1, in agreement with the above coimmunoprecipitation results.

### SFR1 Co-localized with ERα in Nucleus

Both SFR1 and ERα are nuclear proteins [17], [18], [19]. We reasoned that if ERα and SFR1 physically interact, they should be present at the same site in cell nuclei. To determine the intracellular localization of SFR1, an immunofluorescence assay was performed using HeLa cells transiently transfected with FLAG-SFR1 and ER. As expected, transfected SFR1 was present in the nuclei of transfected cells (Figure 1C). No fluorescence signal was detected in control IgG stained cells or in non-transfected cells subjected to the anti-FLAG staining (data not shown). As predicted, confocal immunofluorescence analysis revealed a strong overlap in the expression of FLAG-SFR1 and EGFP-ERα in co-transfected HeLa cells (Figure 1C). Notably, the co-localization between SFR1 and ER was unaffected by E2 treatment (data not shown), further underscoring that their interaction is ligand-independent.

### SFR1 Enhances ERα Transcriptional Activity in Mammalian Cells

**Figure 2:**
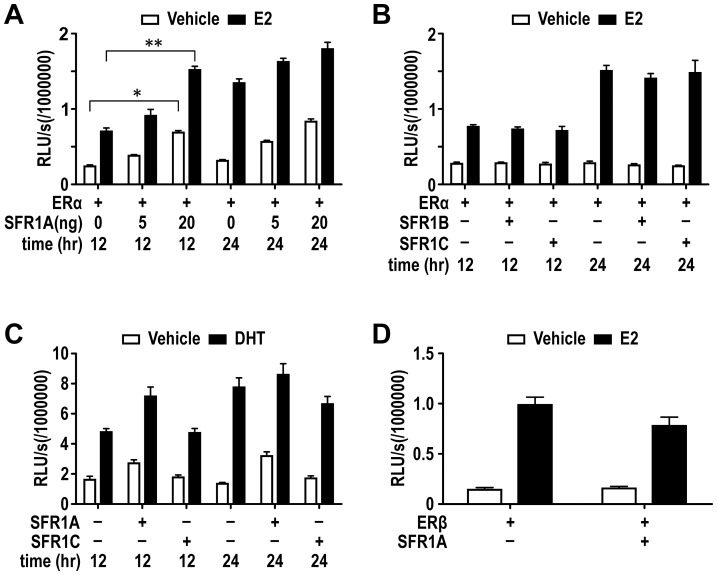
SFR1-A enhances ERα and AR transcriptional activity. The effects of SFR1 overexpression on ERα (A and B), ERβ (D) and AR (C) transcriptional activity were assessed by luciferase reporter assays. Ishikawa cells were co-transfected with nuclear receptors (ERα, ERβ or AR), their respective reporter genes (3xERE-luc and ARE-luc), and indicated amount of p3xFLAG-SFR1-A, p3xFLAG-SFR1-B (20 ng) or p3xFLAG-SFR1-C (20 ng) (D). The cells were treated with vehicle or 1 nM E2 or DHT. Transcriptional activity was measured via luciferase assays at 12 hours and 24 hours after ligand treatments. All luciferase reporter assays were conducted at 12 h after the ligand treatment unless it is indicated in the experiment. 20 ng of SFR1A, SFR1B and SFR1C was transfected unless the amount of DNA was indicated in the figures. All experiments were performed multiple times with triplicate samples. The error bars indicate standard deviations. * = P<0.05; ** = P<0.01; *** = P<0.005.

We have demonstrated that SFR1 interacts and colocalizes with ERα in the cell nuclei. This nuclear interaction of SFR1 and ER raised the question of whether SFR1 can modulate ERα’s transcriptional activity. Therefore, in the following study, we examined the effect of SFR1 and its truncated versions SFR1-B and SFR1-C on ER-mediated transcriptional activity. Transient co-transfection experiments were first conducted in Ishikawa cells that express a low level of SFR1 mRNA, but do not have detectable ER expression (as reflected in our following ERE-luc reporter assays(the cells were originally ER positive, but lost ER expression after several passages of culture). In the ERα-dependent ERE-luc reporter assay, E2 treatment for a period of 12 hrs resulted in a 3-fold activation, which was further increased in a dose-dependent fashion by ectopic expression of SFR1 (Figure 2A). Notably, we also observed a dose-dependent increase of ER ligand-independent transcriptional activity in the presence of SFR1. Similar enhancement was observed in COS-7 cells (data not shown). We then tested whether the truncated forms of SFR1, SFR1-B and SFR1-C, can enhance ERα transcriptional activity in the reporter assay. Little transcriptional enhancement was observed with SFR1-B and C, suggesting that the N-terminus of SFR1 is important for its ER stimulatory function (Figure 2B).

Given that SFR1A can enhance ER transcriptional activity, we asked whether SFR1 can modulate transcriptional activity of ERβ and AR which belong to the same nuclear receptor family. SFR1-A but not SFR1-C enhanced AR transcriptional activity in the presence and absence of DHT (Figure 2C). SFR1, however, did not enhance ERβ’s transcriptional activity in the similar assay (Figure 2D), suggesting the transcriptional enhancement by SFR1 is nuclear hormone receptor-type specific.

### SFR1 is Required for the Transcriptional Activity of Endogenous ERα

**Figure 3:**
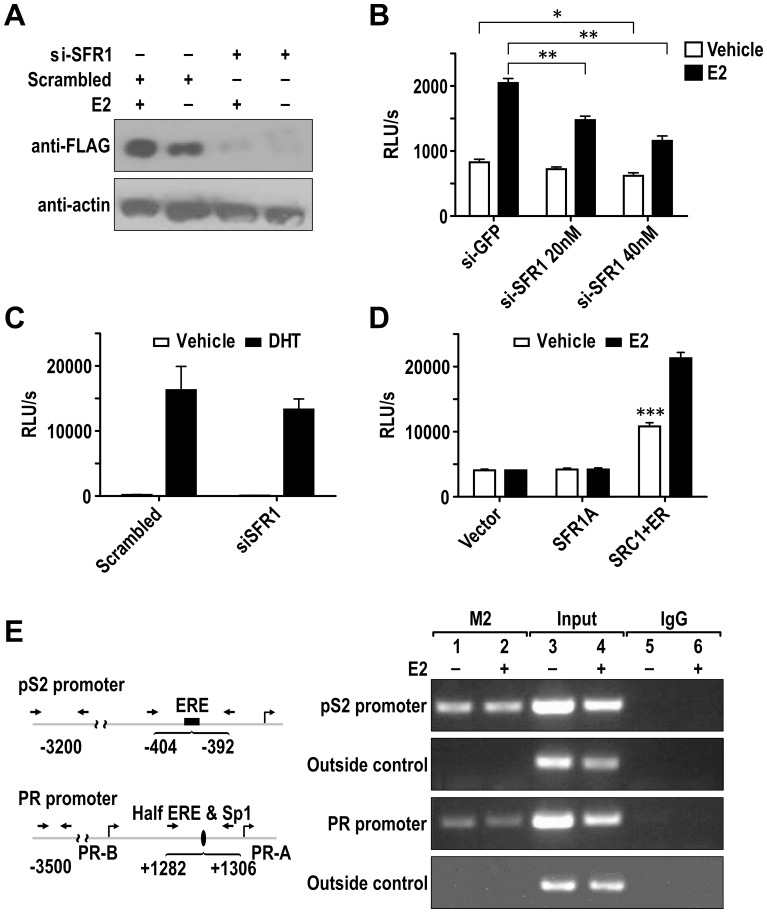
SFR1-A modulates ER transcription activity. (A) Western Blot was used to determine the effect of siRNA of SFR1 and control siRNA on FLAG-SFR1 expression. MCF7 cells were co-transfected with FLAG-SFR1-A along with indicated control or SFR1 specific siRNA (30 nM). Twenty-four hours after the transfection, cells were exposed to E2 (1 nM) or vehicle overnight. Lysates were harvested 48 hrs post-transfection and subjected to Western Blot analysis with the indicated antibodies. (B) Luciferase reporter assay was performed in MCF7 cells to determine the impact of SFR1 knockdown on ER transcriptional activity. MCF7 cells were co-transfected with the indicated amount of SFR1 siRNA and 3xERE-luc reporter or 40 nM scrambled siRNA and 3xERE-luc reporter. 24 hrs after the transfection, cells were treated with 1 nM E2 or vehicle for 24 h. Transcriptional activity was measured via luciferase assay (n = 4). (C) Luciferase reporter assay was performed in LNCaP cells to determine the impact of SFR1 knockdown on AR transcriptional activity. AR-positive LNCaP cells were co-transfected with the indicated amount of SFR1 siRNA and 3xARE-luc reporter or 40 nM scrambled siRNA and 3xARE-luc reporter. 24 hrs after the transfection, cells were treated with 1 nM DHT or vehicle for 24 h. Transcriptional activity was measured via luciferase assay. (D) Mammalian one-hybrid experiment was performed in Ishikawa cells to determine the intrinsic transcriptional activity of SFR1. GAL4DBD (Vector), GAL4DBD-SFR1-A (SFR1-A), and GAL4DBD-SRC1NR-box plus ER (SRC1+ER) were co-transfected with Gal4-luc reporter. The SRC1+ER one-hybrid was used as positive control. The level of transactivation was represented by luciferase activity (n = 4). (E) SFR1-A was recruited to the promoter of endogenous ER target gene promoters. The *in vivo* binding of SFR1-A to pS2 and PR promoters was examined by ChIP assay. FLAG-SFR1-A was transiently transfected into MCF7 cells. Soluble chromatin was prepared from the cells treated with 1 nM E2 (+) for 1 h or vehicle (−) and immunoprecipitated with M2 antibody. Co-precipitated DNA was amplified using primers that flank the ERE in the pS2 promoter region or half ERE and Sp1 site in the PR promoter. The presence of total pS2 and PR promoter DNA in the soluble chromatin prior to immunoprecipitation was included as input. The error bars indicate standard deviations. * = P<0.05; ** = P<0.01; *** = P<0.005.

SFR1 overexpression experiments described above indicated that SFR1 is capable of enhancing both ligand-independent and ligand-dependent transcriptional activity of ERα. The dose-dependent effect of SFR1 indicates that SFR1 is a rate limiting factor in the regulation of ER-dependent transcription. We also showed that ERα and SFR1 physically interact with each other in the cell nucleus. As an extension to the above studies and to further examine the physiological role of SFR1, the requirement of SFR1 proteins for endogenous ER-mediated transcription was examined using RNA interference to deplete endogenous SFR1 in MCF7 cells. We validated the SFR1 siRNA by showing the specific loss of FLAG-SFR1 expression on protein and mRNA levels in SFR1 siRNA-transfected cells (Figure 3A and RT-PCR data not shown). Depletion of SFR1 significantly impaired estrogen-dependent 3xERE-luc reporter activity in MCF7 cells both in the absence and presence of E2 treatment (Figure 3B). GFP siRNA controls showed no effect on ERE-luc activity. This result suggested that SFR1 is required for the optimal level of the transcriptional activity of endogenous ERα.

We also tested whether SFR1 is required for endogenous AR transcriptional activity in LNCaP cells. There was no significant difference of AR transcriptional activity between the control siRNA and SFR1 siRNA transfected cells in the presence of DHT (Figure 3C).

We also asked whether SFR1 harbors an autonomous transcriptional activation domain like that of SRC1. To test this, we fused SFR1 and SFR1C to the heterologous DNA-binding domain of GAL4 and transfected Gal4-SFR1 and Gal4-SFR1C expression plasmid into Ishikawa cells and HeLa cells. The transcription level of a co-transfected luciferase reporter containing four GAL4 binding sites at the promoter region was measured by the luciferase assay. Using GAL4-SRC1-NR-box and ERα one-hybrid as the positive control, neither SFR1 nor the truncated SFR1-C can activate Gal4-luc reporter, indicating that SFR1 does not contain an intrinsic transcriptional activation domain (Figure 3D and data not shown).

Upon ligand stimulation, nuclear receptors including ER recruit coactivators to endogenous target enhancers/promoters *in vivo* to activate target gene transcription [23]. To test whether SFR1 is involved in transcriptional activation of native ERα target genes, we employed chromatin immunoprecipitation (ChIP) assays to look for ERα-dependent recruitment of SFR1 to the ERα binding sites on pS2 and PR promoters in MCF7 breast cancer cells. To facilitate the detection of SFR1, we used MCF7 cells expressing the FLAG-tagged SFR1. Control ChIP assays using normal IgG produced very weak signal. In contrast, SFR1 was detected on both pS2 and PR promoters in the presence and absence of E2 treatment, but not on the outside control region (Figure 3E). To determine the functional significance of SRF1, SRF1 was depleted in MCF7 cells. Depletion of SFR1 abated the expression of pS2 and PR upon estrogen stimulation in MCF7 cells (Fig. S2). Two other ER target genes, Cathepsin D and HSP27, however, did not show significant change upon SFR1 knockdown in MCF7 cells, indicating that SFR1 selectively regulates ER targets. Furthermore, the expression of non-ER target gene, beta-Actin was not significantly changed. Together, these results show that SFR1 is involved in ER-dependent regulation of target genes.

### SFR1 Plays a Role in Estrogen-dependent and Independent Cell Proliferation

**Figure 4:**
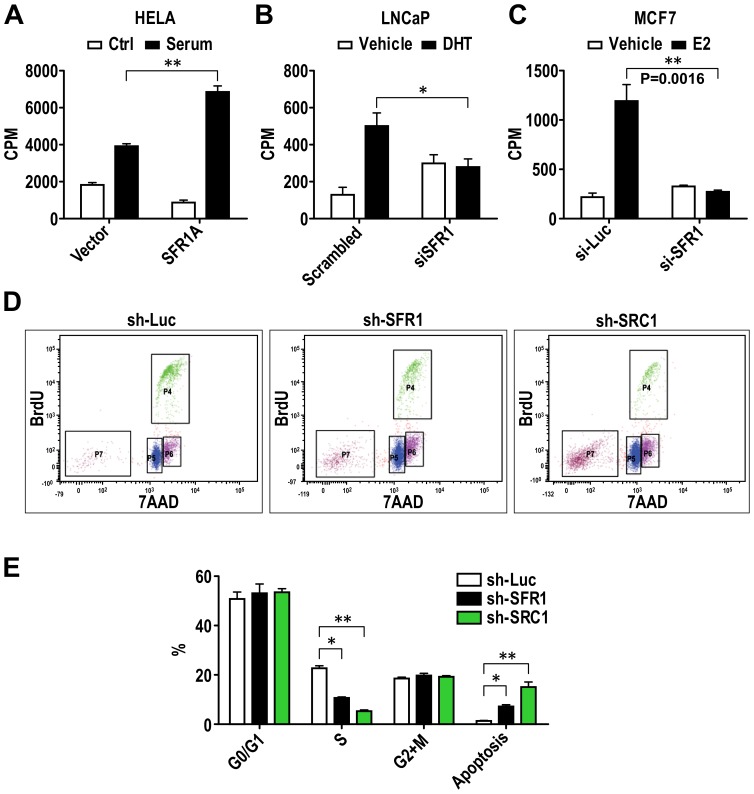
SFR1-A promotes cancer cell proliferation. Thymidine incorporation (A, B and C) and BrdU incorporation assays (D and E) were used to examine the effect of SFR1 on cell proliferation. In thymidine incorporation assays, SFR1-A cDNA was transfected into HeLa cells (A) to determine the impact of SFR1-A on hormone-independent cell proliferation. SFR1 siRNA (40 nM) or scrambled control, were transfected into LNCaP cells (B) and MCF7 cells (C) to determine the effect of endogenous SFR1 protein on ER-dependent cell proliferation. To determine the long-term effect of loss of SFR1 on cancer cell survival and proliferation, SFR1 knockdown MCF7 cell line was established by transducing MCF7 cells with lentivirus expressing sh-SFR1. MCF7 cells expressing sh-SRC1 were used as positive control and MCF7 cells expressing sh-Luciferase (sh-Luc) were established as the negative control. Sh-Luc, sh-SFR1 and sh-SRC1 cells were treated with BrdU for one hour at 37°C and harvested for antibody staining and FACS analysis. FITC-anti-BrdU and 7AAD were used to distinguish cells in G_0_/G_1_ (P5), S (P4), G2/M (P6) and apoptosis phase (P7) in FACS analysis. The results from the FACS analysis were analyzed and plotted with GraphPad Prism5 (E). N ≥3 in all experiments. * = P<0.05; ** = P<0.01; *** = P<0.005.

It is well known that ERα mediates estrogen-stimulated cell proliferation in mammary epithelium. Since SFR1 can enhance ER transcriptional activity, we analyzed its roles in ERα-dependent and -independent cell proliferation. Thymidine incorporation assays were utilized to monitor cell proliferation (as reflected by DNA synthesis) under different conditions. The role of SFR1 in ER-independent cell proliferation was determined in SFR1-transfected HeLa cells. SFR1 significantly enhanced DNA synthesis compared to mock-transfected HeLa cells (Fig. 4A). Because SFR1 RNAi assays indicated that SFR1 is required for ER-mediated transcription in MCF7 cells, we also determined whether the estrogen-dependent growth of MCF7 cells is affected by the depletion of SFR1. As expected, estrogen markedly stimulated the growth of non-transfected (data not shown) and scrambled (control) siRNA-transfected MCF7 cells. However, SFR1 siRNA treatment completely abolished the estrogen-dependent growth of MCF7 cells, suggesting that SFR1 proteins are essential for ER-dependent cell proliferation and raising the possibility that SFR1 could be a therapeutic target in breast cancer (Figure 4C). Similarly, SFR1 knockdown in AR-positive LNCaP cells also blocked AR-dependent cell proliferation (Figure 4B). Furthermore and consistent with the thymidine incorporation data, BrdU incorporation assays in MCF7 cells stably expressing SFR1 shRNA and control luciferase shRNA also showed that SFR1 is required for MCF7 cell proliferation (Figure 4D and E). In SFR1 shRNA-transduced cells, cell apoptosis increased 3 fold (from ∼2% to >6%), and cell proliferation decreased 2.5-fold (a significant decrease of cell numbers in S phase from ∼23.3% to 10.4%). Although the effect of SFR1 knockdown on the cell behavior is qualitatively similar to the effect of knockdown of SRC1 (Fig. 4D), SFR1 shRNA partially released the growth inhibition effect by SRC1 shRNA, indicating that SFR1 may regulate MCF7 cell proliferation in an SRC1-independent fashion (data not shown). Overall, these results indicated that SFR1 plays roles in anti-apoptosis and cell proliferation in cancer cells.

## Discussion

### SFR1 is a Novel ER-interacting Protein and Functions as a Unique Transcriptional Modulator of ER

We have identified the DNA recombination protein SFR1 as a protein that directly binds and regulates ER’s transcriptional activity by multiple complementary studies. First, SFR1 was identified as an ER-interacting protein by the unbiased yeast two-hybrid screen. Second, SFR1 co-localized with ERα in cell nuclei and bound to ER in a ligand-independent fashion. Third, reporter assays showed that the transcriptional regulatory activity of ERα was enhanced by SFR1. Fourth, ChIP assays confirmed the direct recruitment of SFR1 to the ER binding sites of two representative target genes. Finally, not only was SFR1 involved in the endogenous transcriptional activity of ERα, it also enhanced ER-dependent cell proliferation.

Unlike some ER coactivators such as SRC1, no intrinsic transcriptional activity was detected for SFR1. It also lacks sequence homology with other known ER coactivators. These findings, along with the ability of SFR1 to mediate ligand-independent and ligand-dependent interactions with ER, led us to propose that SFR1 functions as a unique transcriptional modulator of ERα. The sequence of SFR1 carries features similar to that of the CoRNR box of N-CoR/SMRT corepressors and that of the NR box of coactivators. CoRNR boxes and NR boxes are known to bind to the ligand-binding domain of nuclear receptors, including ER. These motifs may thus serve as the structural basis of the observed ligand-independent and ligand-dependent associations between SFR1 and ER, indicative of the unique co-regulator function of SFR1. This idea is also consistent with the fact that SFR1 was isolated from a human mammary gland cDNA library on the basis of its interaction with the ERα CDEF domain fragment.

### Mechanism of ER Transcriptional Modulation by SFR1

Although a nuclear localization signal was not predicted for SFR1, its function as a nuclear protein was confirmed by our transient transfection and immunostaining experiments showing SFR1 primarily resides in cell nuclei. SFR1 interacts with ER and enhances both ligand-dependent and ligand-independent ERα transcriptional activity (Figure 2A). However, the enhancement is more robust at 12–16 h after the E2 treatment, suggesting a role for SFR1 in dissociating the co-repressors bound to ERα and/or facilitating the subsequent interaction of ERα with other coactivators to assemble a functional transcriptional complex.

SFR1 may antagonize the function of some ligand-dependent co-repressors, such as ligand-dependent corepressor (LCoR) and repressor of estrogen receptor activity (REA), which bind ERα in the presence of E2 [24], [25], [26]. LCoR inhibits ERα transcriptional activity in a dose-dependent fashion by recruiting co-repressors HDAC3 and CtBP to ERα. REA directly competes with p160 coactivators for binding to ER and can recruit class I and II HDACs [27], [28]. It remains to be determined whether SFR1 acts similarly to CoCoA, a secondary coactivator that binds to GRIP1 with its coiled-coil domain and enhances nuclear receptor transcriptional activity [16].

### SFR1: a Potential New Link between Transcription and DNA Repair and a Therapeutic Target for Breast Cancer

Some transcriptional cofactors can function both in the regulation of transcription and DNA repair, such as the SWI/SNF chromatin remodeling complex, the TFIIH general transcription factor and the DNA-dependent protein kinase (DNA-PK). Our results provide another example of such proteins. Importantly, our work suggests that SFR1 may be specifically involved in ER-dependent regulation of these processes. In this context, Williamson showed that ER is required for an E2-induced DNA double strand break, which is mediated by TopoII-beta [29]. Given that SFR1 binds to ER and that Swi5-SFR1 activates RAD51 recombinase activity [17], [18], it is possible that SFR1 may play an important role in facilitating DNA homologous recombination repair following an ER-mediated DNA double strand break. In a related scenario, recent studies have shown that efficient transcriptional activation by ER requires transient formation of a transcription-induced double strand DNA break [30]. How this transiently-formed DNA break is repaired is not known. Our work raises the intriguing possibility that SFR1 may play a role in this transcription-coupled repairing process.

Like most NR cofactors, SFR1 is expressed in multiple cell types and tissues. A role for SFR1 in the development of mammary tumors is supported by the finding that the mRNA of SFR1 (GenBank: AAH24403) is expressed in mammary tumors of 5-month-old MMTV-Wnt-1 transgenic mice. Further supporting the involvement of SFR1 in the progression of ER-positive breast cancer, we found that SFR1 is required for estrogen-dependent MCF7 cell proliferation.

The effect we observed in proliferation assays is likely contributed by an effect of SFR1 knockdown on cell cycle progression in addition to its role in ER or AR mediated transcription.

While SFR1 may manifest its function in tumors by enhancing ER’s transcriptional activity, given the involvement of SFR1 in the regulation of DNA homologous recombination, it is also possible that hormone receptors, such as ER, may impact the maintenance of genome stability of cells. A further understanding of how SFR1 regulates ER’s transcriptional activity and how ER, through its binding to SFR1, regulates DNA repair, may lead to the identification of important targets that may be used to treat the large number of ER-positive breast cancer patients.

## Materials and Methods

### Plasmids and Antibodies

The cDNA of SFR1 (GenBank accession # BC043256) was purchased from Open Biosystem (Huntsville, AL). SFR1,SFR1-B and SFR1-C cDNA were subcloned into p3XFLAG-7.1 expression vector (Sigma) for FLAG-tagged fusion protein and pM (Clontech) for N-terminal Gal4 DBD fusion protein. pG5-luc reporter construct was a kind gift from Dr. Cong Liu [31]. pSG5AR [32] and Gal4-SRC1-NRbox plasmid containing the nuclear receptor interaction domain of SRC-1 (amino acids 595 to 780) [33] are kind gifts from Dr. Karen Knudsen’s lab. pCMV5-ERα and p3XERE-luc reporter were provided by Dr. Benita Katzenellenbogen (University of Illinois). Anti-FLAG M2 antibody is from Sigma (Cat # F1804). Anti- ER H184 polyclonal antibody is from Santa Cruz (Cat # sc-7207). Goat-anti-rabbit secondary antibody (Cat # 31460) and goat-anti-mouse secondary antibodies (Cat # 31430) are from Pierce. Mouse monoclonal anti-beta actin antibody was from Sigma-Aldrich (Cat # A3853). Alexa Fluor 568 anti-mouse secondary was from Molecular Probes. FITC anti-BrdU was from BD Bioscience (Cat # 347583).

### Cell Culture and Transient Transfection

Ishikawa cells were grown in MEM (Fisher) with 10% fetal bovine serum (Hyclone). HeLa and MCF7 cells were grown in DMEM (Fisher) with 10% fetal bovine serum. The transfections were carried out using SilentFect (Bio-Rad) or Lipofectamine 2000 (Invitrogen) for siRNA experiments, or with Fugene 6 (Roche) for plasmids only, according to the manufacturers’ protocols. At 20 h post-transfection, the cells were treated with specific ligands or vehicle (ethanol) and were harvested after 18–24 h of incubation. The cells were lysed in reporter lysis buffer (Promega) and assayed for luciferase activity.

### Chromatin Immunoprecipitation (ChIP)

ChIP assay was performed as previously described [23]. The PCR primers used were as follows:

pS2 Forward: 5′TTA GCT TAG GCC TAG ACG GAA TGG; pS2 BACKWARD: GAC GAC ATG TGG TGA GGT CAT CTT-3′; pS2 OUTSIDE Forward: GGG GCT GTT TTC CTG TGT TA-3′; pS2 OUTSIDE BACKWARD: CAT TTG GGC CTA TCT GGA TG-3′; PR Forward: AAA GGG GAG TCC AGT CGT CA-3′;PR BACKWARD: CTG GTCCTGCGTCTTTTCGT-3′; PR OUTSIDE Forward: GGA AGG TTA GAG GAA GAA TG-3′; PR OUTSIDE BACKWARD: CCT TTG CAC CTT TGT TGA GA-3′.

### Thymidine Incorporation

The assay was performed in LNCaP, HeLa and MCF7 cells as previously described [34].

### Reverse Transcription-PCR

For RT-PCR, total RNA was extracted and purified from cells with Trizol (Invitrogen). Reverse transcription reactions were carried out with the Retroscript kit (Ambion Cat # 1710), following the manufacturer’s protocol. The resulting products were subjected to PCR amplification with the following SFR1 primers: Forward: 5′ GCT GAA AAA GCC AAA TTG GTG 3′and Backward: 5′ GCT GGC TAC AGC TTC TCC ACT 3′.

### RNA Interference

SFR1 siRNA (Dharmacon, Smart mix) or the SFR1 siRNA and 3XERE-luc reporter plasmid were introduced into MCF7 cells in 96-well or 12-well plates with SilentFect (Bio-Rad Cat # 170-3360) or Lipofectamine 2000 (Invitrogen), respectively, following the manufacturers’ protocols. Luciferase activities were assayed with Luciferase Reporter Assay System (Promega).

### Co-immunoprecipitation and Western Blot

Co-IP was performed according to the standard protocol provided by Santa Cruz Biotechnology. Western blot was performed according to the standard protocol provided by Sigma for anti-FLAG M2 antibody. Nitrocellulose membrane was from Bio-Rad (Cat # 162-0094). ECL kit is from Amersham Biosciences (Cat # 1059250).

### Immunofluorescence Assay

The immunofluorescence assays were performed according to the standard protocol provided by Sigma for anti-FLAG M2 antibody.

### Digital Photography

The pictures were photographed with a Zeiss LSM510 confocal microscope (Carl Zeiss Co. NY) with 63X Zeiss objective lens.

### Mammalian Hybrid Assays

Mammalian hybrid assays were performed as described [35], except that a pM-SRC1 construct and pCMV5ERα were co-transfected with 4XGal4-luc reporter into the Ishikawa cells. 16 h after the transfection, the cells were treated with E2 for 24 h and subjected to luciferase assay.

### Sh-SFR1 Cell Line

MCF7 cells were transduced with pLK0.1-shSFR1 lentivirus (Open Biosystem) and selected with 2 µg/ml puromycin until colonies formed. The puromycin-resistant clones were selected and expanded for different assays.

### BrdU FACS Analysis

BrdU FACS analysis was performed as described [36] with modifications. Prior to harvesting, cells were pulsed with BrdU for 1 hour. Cells were harvested by trypsinization and fixed in 70% EtOH overnight at 4°C. Cell pellets were washed once with IFA buffer (1x DPBS (Invitrogen), 4% FBS and 0.1% NaN3) and once with IFA +0.5% Tween 20 prior to 1 hour incubation in FITC-conjugated anti-BrdU (BD Pharmingen, 15 µl in 100 µl IFA) at room temperature. Following incubation with antibody, cells were washed once in IFA +0.5% Tween 20 and incubated with 7AAD (BD Pharmingen). Cells were sorted by the FACS Canto flow cytometer (BD). Histograms/scatter plots represent 20,000 gated events. Cell cycle analysis was performed using FACS Diva software. BrdU data are represented as percent of untreated control.

### Statistical Analysis

Data were analyzed and plotted using Prism 5.0 (GraphPad Software). Statistical analysis was done with student t test for all analyses. A value of P<.05 was considered significant.

## Supporting Information

Figure S1 SFR1 protein structure and mRNA expression in different tissues and cells. (A) Schematic representation of SFR1 protein isoforms. The NR-box-like, CoRNR-like and Coiled-coil domains are indicated. Existence of the Coiled-coil domain was predicted using ELM (www.expasy.com). (B) SFR1-A is conserved in mammals. The multiple sequence alignment was performed with EMBL-EBI ClustalW online software: http://www.ebi.ac.uk/clustalw/. The homology of human SFR1-A with other mammalian species is shown, with asterisks indicating positions of identical amino acids, and dots and colons indicating the positions of similar amino acids. CoRNR-like and LXXLI motifs are indicted by rectangular boxes. (C) SFR1 mRNA is expressed in various cell lines and tissues. Total RNA was isolated from cell lines and subjected to RT-PCR with SFR1 primers. Human fetal brain and skeletal muscle cDNA were analyzed as well. Lanes: 1. Ishikawa cells; 2. C4-12 cells; 3. MCF7 cells; 4. Human Skeletal Muscle cDNA; 5. Human Fetal Brain cDNA; 6. Mouse embryonic fibroblast (MEF) cells. C4-12 and MCF7 are human breast cancer cell lines. Ishikawa cells are human endometrial cancer cells. The human SFR1 primers do not amplify mouse SFR1.
(TIF)

Figure S2 SFR1 is required for the expression of ER target genes. qPCR was performed on RNA isolated from MCF7 cells transfected with siSFR1 or siGFP (control). 24 hrs after the transfection, cells were treated with E2 or vehicle overnight for determining mRNA expression of Progesterone receptor (PR),pS2, Cathepsin D (CTSD), Heat Shock Protein 27 (HSP27), and beta-Actin. The expression of different genes was normalized to GAPDH. All assays were performed in triplicates and error bars represent Standard Deviation (** = P<0.01, ns = no significance). Data are representative of three independent experiments.
(TIF)
