# Supplementary figures and images for: DNA Homologous Recombination Factor SFR1 Physically and Functionally Interacts with Estrogen Receptor Alpha
Source: PLoS One. 2013 Jul 9;8(7):e68075. doi: 10.1371/journal.pone.0068075 (PMC3706619; doi:10.1371/journal.pone.0068075)

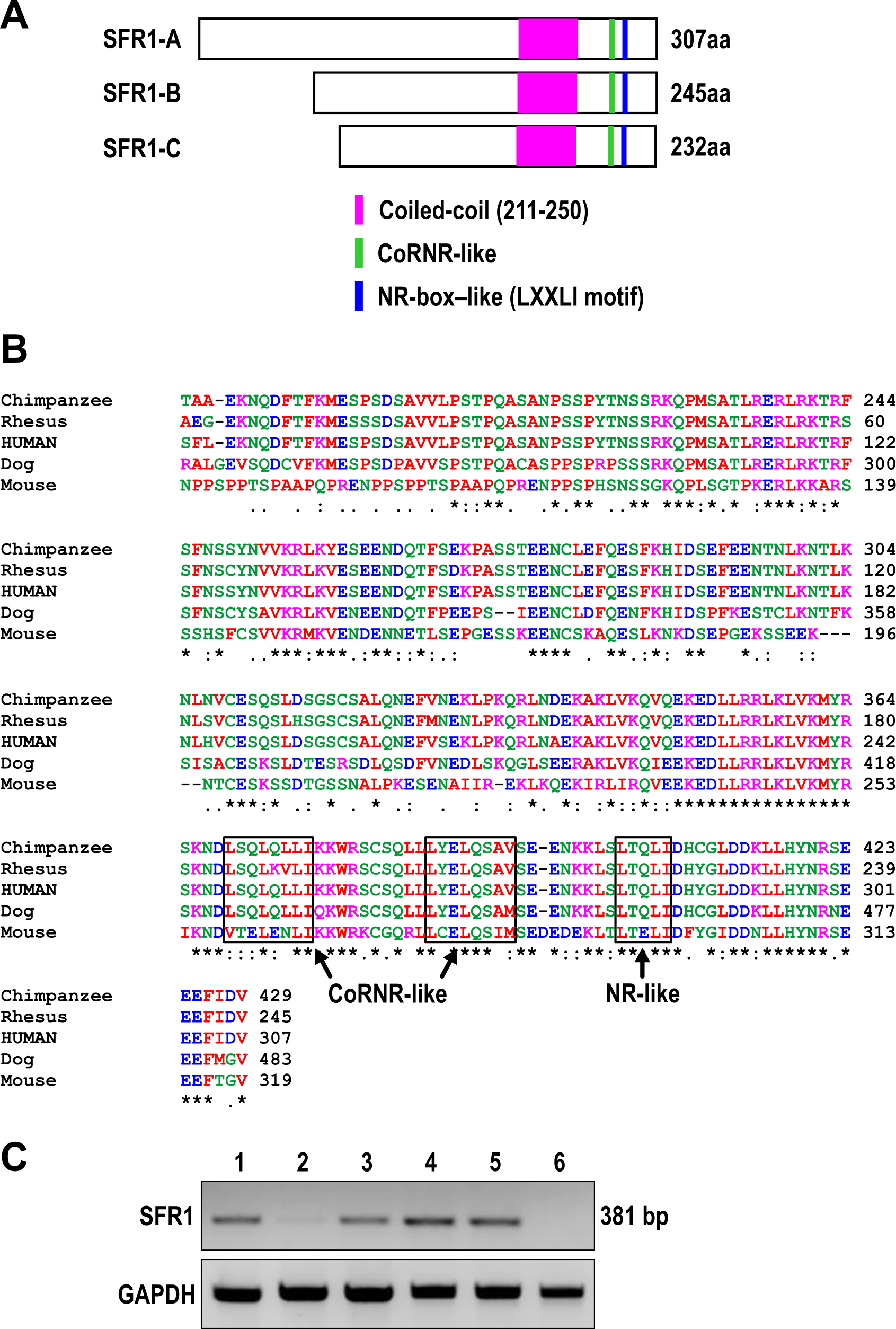

Supplement: Figure S1 — SFR1 protein structure and mRNA expression in different tissues and cells. (A) Schematic representation of SFR1 protein isoforms. The NR-box-like, CoRNR-like and Coiled-coil domains are indicated. Existence of the Coiled-coil domain was predicted using ELM (www.expasy.com). (B) SFR1-A is conserved in mammals. The multiple sequence alignment was performed with EMBL-EBI ClustalW online software: http://www.ebi.ac.uk/clustalw/. The homology of human SFR1-A with other mammalian species is shown, with asterisks indicating positions of identical amino acids, and dots and colons indicating the positions of similar amino acids. CoRNR-like and LXXLI motifs are indicted by rectangular boxes. (C) SFR1 mRNA is expressed in various cell lines and tissues. Total RNA was isolated from cell lines and subjected to RT-PCR with SFR1 primers. Human fetal brain and skeletal muscle cDNA were analyzed as well. Lanes: 1. Ishikawa cells; 2. C4-12 cells; 3. MCF7 cells; 4. Human Skeletal Muscle cDNA; 5. Human Fetal Brain cDNA; 6. Mouse embryonic fibroblast (MEF) cells. C4-12 and MCF7 are human breast cancer cell lines. Ishikawa cells are human endometrial cancer cells. The human SFR1 primers do not amplify mouse SFR1. (TIF) [file pone.0068075.s001.tif]

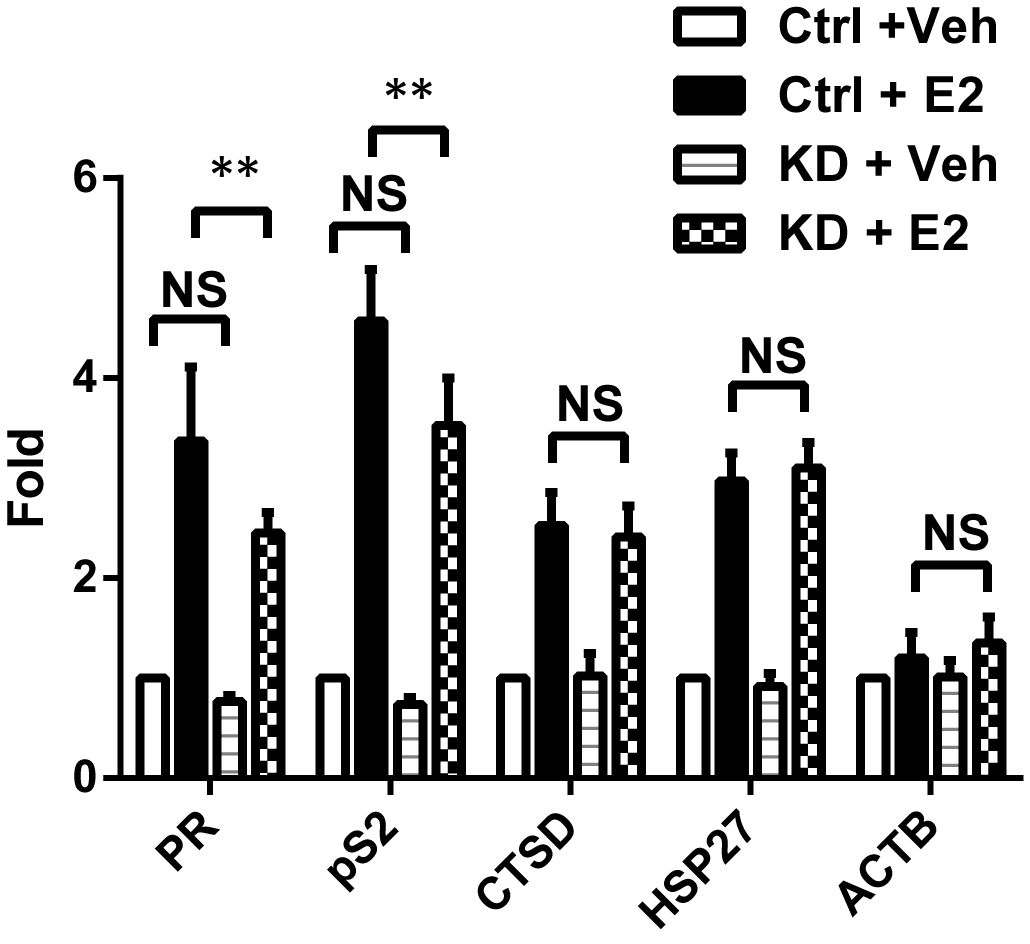

Supplement: Figure S2 — SFR1 is required for the expression of ER target genes. qPCR was performed on RNA isolated from MCF7 cells transfected with siSFR1 or siGFP (control). 24 hrs after the transfection, cells were treated with E2 or vehicle overnight for determining mRNA expression of Progesterone receptor (PR),pS2, Cathepsin D (CTSD), Heat Shock Protein 27 (HSP27), and beta-Actin. The expression of different genes was normalized to GAPDH. All assays were performed in triplicates and error bars represent Standard Deviation (** = P<0.01, ns = no significance). Data are representative of three independent experiments. (TIF) [file pone.0068075.s002.tif]
